# Hyperuricemia-associated Mesoamerican Nephropathy: Case Report and Review of Literature

**DOI:** 10.7759/cureus.3282

**Published:** 2018-09-11

**Authors:** Adebayo Atanda, Janna Henry, Gbeminiyi Samuel, Olusayo Fadiran, Curtis Frederick, Oluwatosin Omole, Kolapo Idowu, C C Mere

**Affiliations:** 1 Internal Medicine, Howard University Hospital, Washington, D.C., USA; 2 Internal Medicine, Howard University Hospital, Hyattsville, USA; 3 Medicine, Howard University Hospital, Washington, D.C., USA; 4 Family Medicine, Howard University Hospital, Washington, D.C., USA; 5 Family Medicine, Howard University Hospital, Orlando, USA; 6 Nephrology Unit, Howard University Hospital, Washington, D.C., USA

**Keywords:** gout, mesoamerican nepropathy, ckd, hyperuricemia, nephropathy, aki, uric acid

## Abstract

Mesoamerican nephropathy (MeN), formerly called chronic kidney disease of unknown cause (CKDu), refers to chronic kidney disease (CKD) that presents in young and middle-aged Central Americans in the absence of any clear etiology. MeN is a relatively new diagnosis with rapidly rising prevalence in specific regions of El Salvador and Nicaragua, Guatemala, and Costa Rica. It is seldom associated with hyperuricemia. We present a case of a patient with gouty arthritis and hyperuricemia with associated CKD due to MeN. We also provide a review of literature of this disease.

## Introduction

Mesoamerican nephropathy (MeN) was first described in El Salvadorian patients when it was noted that a high percentage of patients were initiated on dialysis with no possible etiology of chronic kidney disease (CKD) [1-3]. Various risk factors have been described including agricultural and physically demanding work, pesticide. Nonsteroidal anti-inflammatory drugs (NSAIDs) and lower socioeconomic class are also important underlying risk factors for MeN [4,5]. There is a paucity of reported cases of hyperuricemia-associated MeN. We report a 41-year-old man with this condition and delved into a thorough review of literature on this relatively novel disease.

## Case presentation

We report a 41-year-old man with history of hyperuricemia and gouty arthritis who presented with progressive dyspnea of three days duration. The patient endorsed multiple painful swelling in his hands and elbow with limitation of motion. He also mentioned new lesions in his ear lobes. The patient is a former smoker with 10 pack year history. He worked as agricultural field laborer and truck driver in El Salvador for six years before immigrating to the United States. Review of system is positive for nocturia.

**Figure 1:**
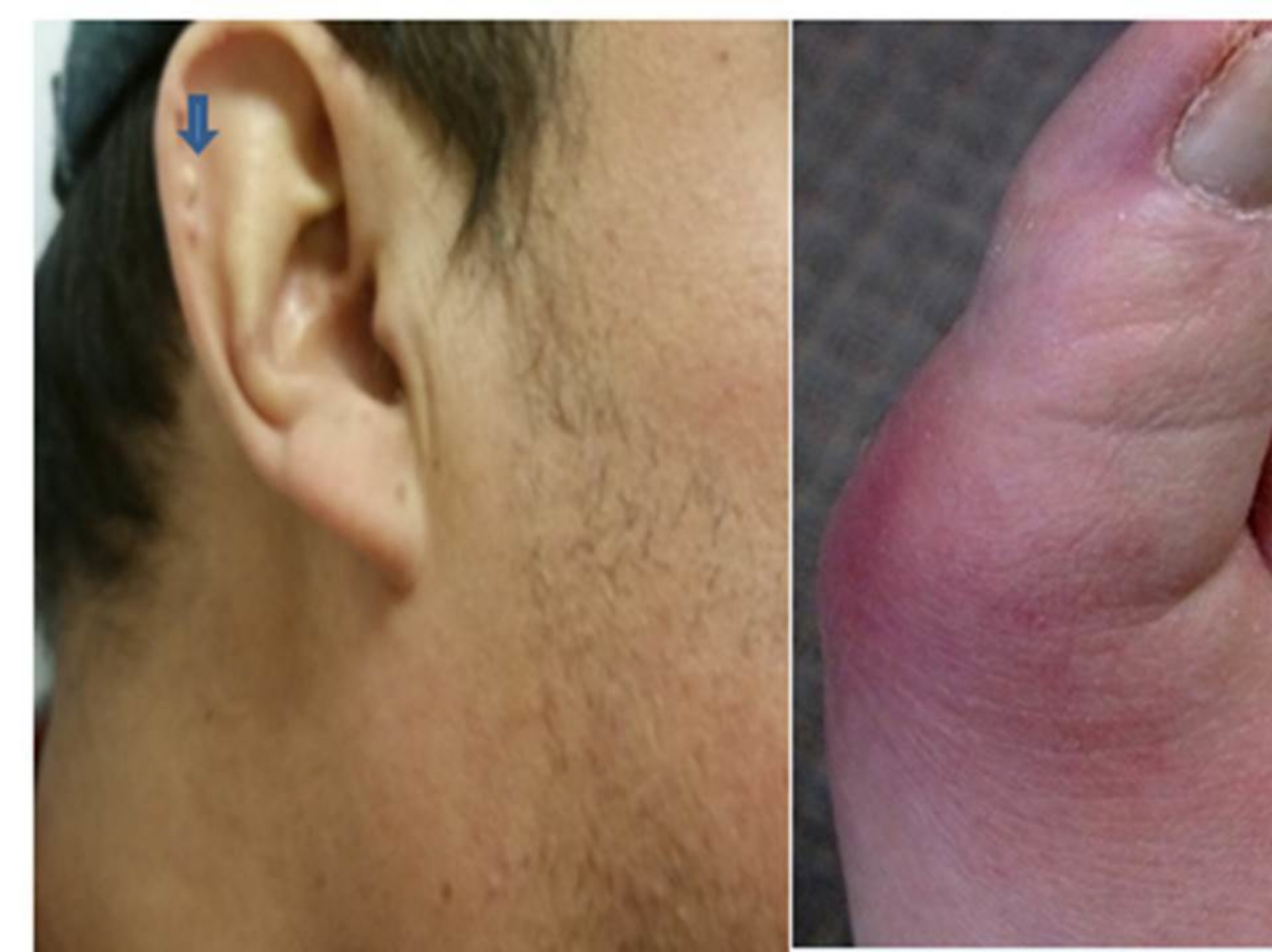
Left: Right Pinna Tophi (blue arrow); Right: Gouty flare of right first metatarsophalangeal joint.

Examination revealed pale and icteric gentleman with yellowish deposits in both ear lobes (Figure 1). Auscultation revealed bi-basilar fine crackles. Also, the patient had joint swelling and deformity associated with hotness and redness in his right elbow, right and left hand, proximal interphalangeal joint, distal interphalangeal joint, and metacarpophalangeal joint (Figure 1). Swellings were mildly tender to palpation. Important labs on admission include blood urea nitrogen (BUN) of 65, creatinine of 8.6 and hemoglobin of 6.8. Extensive laboratory and radiologic investigations for causative factors for CKD were negative. He was subsequently started on hemodialysis following worsening renal function. During the course of admission, he was treated for an acute flare of gouty arthritis of his right great toe with renally dosed colchicine. Also, an arterio-venous fistula was secured before discharge.​​​​​​​

## Discussion

MeN, formerly called CKDu, refers to CKD that presents in young, agricultural workers in Central America and other tropical parts of the world, in the absence of any clear etiology [4,5]. Most patients have a history of physically demanding agricultural work in a hot climate [6].

Although the etiology of MeN is still unclear, hard physical work in hot climates appears to be the most important underlying risk factor [4,5]. High temperatures predispose to dehydration, volume depletion, and loss of minerals and may precipitate rhabdomyolysis. Prolonged working under high temperature may result in cyclic uricosuria in which uric acid concentrations exceed solubility, leading to the formation of dihydrate urate crystals and local injury from the direct cytotoxic effect of the increased urinary uric acid on renal tubules [1,6]. Another proposed mechanism for hyperuricemia causing CKD is the ability of uric acid to induce glomerular hypertension.

It is postulated that the most likely cause of MeN is repeated episodes of acute kidney injury (AKI) related to dehydration, loss of minerals, hypovolemia sometimes accompanied by rhabdomyolysis, systemic inflammation, use of NSAIDs, and oxidative injury [1,2]. CKD may arise from repeated episodes of AKI.

Additionally, excess use of NSAIDs may contribute to MeN [2]. Perturbations in the renin-angiotensin system (RAS) due to excessive and repeated losses of salts due to excessive sweating may also be involved in the pathogenesis [7]. Activation of the polyol pathway and increased renal cortical fructose levels during episodes of dehydration may likewise contribute to MeN [4].

Diagnosis of MeN should be considered in at-risk patients who present with a decreased estimated glomerular filtration rate (eGFR) without proteinuria or hematuria and without other causes of CKD [8].

The management of MeN is supportive care involving treatment of signs and symptoms and prevention of progression. Prevention of onset and progression of MeN includes advising patients to drink sodium- and potassium-containing fluids [7]. It has been suggested that unlike in other CKD, MeN patients should avoid angiotensin-converting enzyme (ACE) inhibitors or angiotensin receptor blockers (ARBs). This is because ACE inhibitors and ARBs may result in blockade of the RAS and may predispose individuals to AKI secondary to hypovolemia, subsequently increasing the risk of MeN. The avoidance of sugary beverages and fructose-containing fluids has also been suggested to reduce onset and progression of MeN [7].

## Conclusions

In the face of global warming and increasing number of unexplained CKD cases, there is a need for a high index of suspicion for MeN in patients who live or grew up in the tropics presenting with CKD especially with investigation findings revealing no abnormality. Also, identifying and instituting appropriate preventive lifestyle pattern could prevent the onset and potentially slow down the progression of MeN.
